# Family-based treatment with transition age youth with anorexia nervosa: a qualitative summary of application in clinical practice

**DOI:** 10.1186/s40337-015-0037-3

**Published:** 2015-02-01

**Authors:** Gina Dimitropoulos, Victoria E Freeman, Brooke Allemang, Jennifer Couturier, Gail McVey, James Lock, Daniel Le Grange

**Affiliations:** Factor-Inwentash Faculty of Social Work, University of Toronto, Toronto, Ontario Canada; Eating Disorders Program, University Health Network, 200 Elizabeth Street, Toronto, ON M5G 2C4 Canada; Sickle Cell and Thalassemia Transition Clinic, The Hospital for Sick children, Toronto, ON Canada; Child and Adolescent Psychiatry, McMaster Children’s Hospital, Hamilton, Canada; Community Health Systems Resource Group, The Hospital for Sick Children, Toronto, ON Canada; Ontario Community Outreach Program for Eating Disorders, Toronto, ON Canada; Department of Psychiatry and Behavioral Sciences, Stanford School of Medicine, Stanford, CA USA; Eating Disorders Program Department of Psychiatry and Department of Pediatrics, University of California, San Francisco, CA USA

**Keywords:** Anorexia nervosa, Clinicians, Family-based treatment, Qualitative research, Transition, Transition age youth, Family support, Adolescents, Parents, Developmental stage

## Abstract

**Background:**

Family based treatment (FBT) has been empirically investigated in adolescents between the ages of 12 and 19 years of age. Although parental control over eating symptoms and the weight gain process are temporary and necessary due to serious medical complications, FBT may be developmentally inappropriate when working with older adolescents. To date, there are no studies identifying how the principles of this model are used differentially across different stages of adolescence. This study aimed to identify how clinicians informed by FBT employ this model with transition age youth (TAY) (16–21) with an eating disorder.

**Methods:**

Using content analysis, seven individual interviews and six focus groups were conducted with 34 clinicians from specialized Eating Disorder Treatment programs across Ontario, Canada.

**Results:**

Participants consistently reported modifying FBT to increase its developmental appropriateness with TAY in the following ways: working more collaboratively with the patient, increasing individual time spent with the patient prior to the family meeting, providing greater opportunities for the individual to practice eating without parental support and introducing relapse prevention in the latter phase of the treatment.

**Conclusions:**

In all adaptations of the model, participants in focus groups and individual interviews cited the age of the individual with the eating disorder, their level of autonomy and independence in all areas of their lives, and their pending transfer of care from paediatric to adult eating disorder programs as main factors that influenced the modification of FBT with TAY. While adaptations were made across all three phases of FBT, adherence to the model progressively declined over the course of treatment with adaptations increasing significantly in the later phases. Future research is needed to evaluate the effectiveness of an adapted version of FBT with TAY.

## Background

Family-Based Treatment (FBT) is an outpatient intervention for adolescents with eating disorders that emphasizes parent involvement in addressing eating disorder symptoms, while also promoting parental responsibility for facilitating treatment adherence [1-4]. FBT has been empirically tested with adolescents and adults with eating disorders [5,6] and consists of three phases. In the first phase, parents remain chiefly responsible for the management of the illness and for facilitating behavioural changes. A fundamental principle of FBT is the externalization of the illness. Externalization, a concept first described by Michael White [7] in the context of FBT refers to separation of the individual from the eating disorder throughout treatment in order to reduce parental blame and promote parental empowerment. For instance, by assigning a name to the illness, parents are encouraged to work together as a team against the eating disorder rather than their child. Parental efforts are directed towards undermining the power of the eating disorder over their child. As the symptoms remit (weight restoration and/or amelioration of binge eating and purging), the focus shifts to gradually allocating more responsibility for eating, weight gain and reduction in bulimic behaviours from the parents to the adolescent. Clinicians must work diligently to ensure that parents do not relinquish control over eating to an adolescent who may be struggling with significant fears of eating a variety of food and reluctant to maintain a normal weight. In the final phase, the therapist shifts discussions to normal adolescent developmental issues that may interfere with recovery.

FBT is efficacious in achieving weight restoration in adolescents with a short illness duration who are medically stable [8,9]. For adolescents with anorexia nervosa (AN), FBT is superior to individual therapy at 1 and 5-year follow up [5,6] and when compared to individual adolescent-focused therapy, FBT is shown to be more effective at achieving weight restoration [3,8] and reducing rates of hospitalization [9,10]. However, an earlier study suggests that for those with AN, FBT may be less effective with older adolescents and/or when the illness develops a chronic course [5,9].

The results from two randomized controlled trials (RCTs) of FBT in bulimia nervosa (BN) [11-13] also reveal positive treatment outcomes. Although the content of the family therapy differed from the FBT established by Lock and Le Grange [2], Schmidt et al. [13] compared family therapy to CBT-Guided Self-care for adolescents with BN and found no differences at a 6-month follow up. In the other RCT, FBT for adolescents with BN was more effective at the end of treatment and at 6 months follow-up in reducing binge eating and purging behaviours compared to individual supportive psychotherapy [11]. Finally, a recent meta-analysis reviewing the literature on the use of FBT with both AN and BN revealed that FBT is more effective than individual therapy at 6 to12 months post treatment for both disorders [14]. Overall, preliminary findings on the effectiveness of FBT indicate good outcomes for adolescents suffering from AN with a short duration of illness and for adolescent patients with BN.

Despite empirical evidence supporting the effectiveness of FBT as the first line of treatment for adolescents with AN or BN, dissemination studies show that clinicians do not adhere closely to the principles and phases of the model in routine clinical practice [15,16]. These studies did not specifically inquire if fidelity to FBT was influenced by the empirical research that indicates this model may work less effectively with older adolescents with a longer illness course [5,9]. Few studies have systemically investigated if and how clinicians use the principles and interventions of FBT with individuals across the developmental stages of adolescence.

To date, the age of adolescents in the randomized controlled trials described above ranged from 12 to 18 years of age. However, manualized FBT has been developed and recommended for individuals with eating disorders up to the age of 19 years [2,17,18]. Although parental control of eating symptoms is temporary and the model explicitly recommends returning control back to the adolescent when problematic eating behaviours have remitted, there may be unique therapeutic challenges to using this model with transition age youth. Transition age youth (TAY) encompass young people between the ages of 16 to 25 who are undergoing major life transitions including individuating from their family, transitioning from secondary to post-secondary school, and transferring out of paediatric to adult health care systems that are likely to rely on adult versus family centred care models [19-22]. The emphasis on parental involvement in re-nourishing their child to health in FBT may be challenging to implement when individual autonomy particularly in older adolescents is highly valued in most Western societies. In a qualitative study [23], paediatric clinicians described experiencing clinical tension when working with transition age youth; that is, clinicians understood the drive for greater autonomous decision making in TAY, but also recognized how prolonged starvation and malnourishment hinders them from managing the illness and recovery without parental involvement. In recognition that the foundational models of FBT may require some modifications to increase acceptability to TAY, Chen et al. [24] slightly adapted the original model by emphasizing greater collaboration between the patients and their parents. This adapted model was evaluated in a small case series on four TAY outpatients (age 18–21) with AN with a duration of illness ranging from one to three and a half years. Findings from this case series demonstrated weight restoration and a return of menses in three out of four participants.

The rationale underlying this study is based on two key considerations. Firstly, despite the fact that FBT is the leading evidence-based treatment for AN, it works better in younger adolescents as opposed to TAY. Second, we propose that an important reason for this difference is the developmental progression during late adolescence/early adulthood to an increasing desire and need for autonomy. This comes into direct conflict with the emphasis in FBT on parents taking control of the adolescent’s eating and meal routines. Parental involvement in assisting with monitoring meals may seem inappropriate for TAY who have begun the process of achieving greater independence from their family, who increasingly rely on their peers for emotional support, and are launching from secondary to post-secondary education or new employment opportunities. We are interested in identifying how FBT can be adapted for TAY, maintaining the core features of the treatment while being sensitive to developmental factors. For instance, it may be that the technique of externalization is more difficult to use in older compared to younger adolescents, as has been discussed with other mental health disorders [25]. As a first step, we conducted focus groups and individual interviews of clinicians to glean insights as to how FBT is already being adapted in young people age 16–21 (the age range is consistent with Chen et al [24]). The chief objective of this exploratory study was to identify if FBT informed clinicians use this treatment model differentially in this age group and what adaptations, if any, are made to this model for TAY. A secondary objective was to obtain clinical impressions of how manualized FBT may be modified for older adolescents and young adults. The findings regarding the secondary objective will be reported in another manuscript.

## Methods

### Design

Directed content analysis was used to establish an understanding of how clinicians use FBT with individuals across the developmental phase of adolescence with a particular focus on TAY. The chief objective of directed content analysis is to validate and extend an existing theoretical model and/or empirical findings about phenomena of interest [26]. As we premised this study on a theoretical model (that a family based treatment model is appropriate for TAY but requires adaptations to better fit the developmental needs of this age group), directed content analysis was chosen over other methods of analysis such as conventional content analysis which seeks to create a theory by starting with the data. Directed content analysis was utilized to extend the family based treatment model by systematically inquiring how each phase and intervention is applied by trained clinicians for TAY with anorexia nervosa. The FBT model provided a framework to assign codes to the variables of interest.

This study utilized two different qualitative data collection procedures [27]. One-on-one interviews were conducted with participant clinicians who had received training and/or certification in FBT (please see inclusion criteria below). A focus group methodology was utilized with paediatric teams who identified manualized family-based treatment as the dominant modality in their program. Individual interviews were employed to glean detailed descriptions from clinician participants regarding their use of manualized FBT with families of individuals across the developmental phase of adolescence. In contrast to the interviews, focus groups were employed for the purpose of capturing the rich discussions hearing a range of perspectives from inter-disciplinary teams about the topic of interest [28-32]. Although the level of experience in FBT varied in the focus groups, the composition of the focus groups was otherwise homogenous (whole paediatric teams).

### Recruitment procedures

A purposive sample of clinicians and paediatric eating disorder programs providing treatment to children and adolescents with eating disorders were recruited in Ontario, Canada. We created specific criteria to target publicly funded tertiary eating disorder programs in Ontario that employed family based treatments. Given the objective of the study, we could not rely on a convenience or random sample since we specifically aimed to obtain information regarding the experiences of using FBT from a variety of programs whose composition and access to resources differ. We were interested in understanding whether multidisciplinary teams work differently with families of individuals with anorexia nervosa depending on the phase of adolescence. We specifically targeted multidisciplinary teams since adolescents and TAY with eating disorders typically receive treatment in specialized eating disorder programs in which multidisciplinary teams are the norm. Secondly, we sought to recruit paediatric teams from across the province of Ontario to ensure that the findings represented different geographic regions (rural versus urban) and varying levels of resources (smaller versus larger paediatric programs). To recruit clinicians and clinical teams, the Director of the Ontario Community Outreach Program for Eating Disorders (OCOPED), a publicly funded eating disorder treatment training program (www.ocoped.ca), disseminated information about the study via email to managers and clinicians working within adolescent eating disorder programs in Ontario, Canada. OCOPED oversees the training of clinicians who are employed in specialized inpatient, day treatment and outpatient programs for eating disorders; it includes treatment programs in urban, rural and remote areas. Clinicians interested in participating in the study were invited to contact one of the researchers via telephone to arrange an interview or a focus group. For the purposes of this study, inclusion criteria for clinicians and/or teams were as follows: 1) affiliated with a provincial network of publicly funded, specialized eating disorder treatment programs; and, 2) clinicians and/or teams identified as using FBT in their clinical work with adolescents. The following exclusion criteria were employed: clinicians and/or teams from paediatric eating disorder programs not using FBT; adult eating disorder programs; and private practitioners.

### Data collection

All participants provided informed consent and this study received approval from the University Health Network Research Ethics Board, Toronto, Canada. Two methods were used to obtain information in this qualitative study. First, paediatric eating disorder teams were asked to participate in a 60 to 90 minute Focus Group (FG) led by the principal investigator (GD) and attended by at least one other member from the research team. Of 12 paediatric programs in Ontario, six met our inclusion/exclusion criteria and all of these sites participated. The remaining six sites did not meet our inclusion/exclusion criteria. Specifically, these six sites included four sites that did not have training in this model because they were a newer program with insufficient resources or no opportunities for training in FBT, and two sites that had adopted different treatment modalities. Second, we identified individuals in training or who were FBT certified therapists to participate in an individual interview. These individuals were identified through multiple sources, including: 1) Directors of the participating Paediatric Eating Disorder Programs, 2) Website for FBT certified therapists, 3) Self-identified, and, 4) Director of the Ontario Community Outreach Program for Eating Disorders. The individual interviews offered us invaluable information on how the specific interventions and the application of manualized FBT were being applied by therapists who had more extensive training in this model. We were unable to ascertain with accuracy the number of clinicians in Ontario receiving training in FBT and therefore cannot state how clinicians who took part in the study are different from the ones who did not participate.

Clinicians involved in the study identified personal demographics as well as their level of FBT training via a self-report form and were categorized into four levels of expertise: 0 refers to no training or purposeful exposure to FBT, 1 for no formal training but purposeful exposure to FBT through readings, colleagues, or brief seminars, 2 refers to individuals in the process of becoming FBT certified and 3 includes only FBT certified clinicians as described by the training institute for this model (Table 1). All clinicians who took part in individual interviews identified as being level 2 or 3 while clinicians from the FGs identified along the spectrum of 0 to 3. Each FG was comprised of members from clinical teams treating adolescent eating disorders and identified as using FBT as the dominant model in their treatment practices and program. All focus groups and individual interviews were conducted at the participant’s place of employment. The objective of the FGs and interviews was to investigate whether FBT is applied differentially depending on the age of the patient. A structured interview guide was administered by the PI (GD) with probes to elicit responses on the use of FBT with families of TAY (Table 2). The second member of the research team in attendance took field notes on significant processes and observations throughout the FGs that warranted further discussion. The research team met after each interview and FG to review and document their impressions and observations of the process and content of the focus group. We also reflected on commonalities (and any new material) emerging across focus groups and documented this process as well. The research team also agreed when saturation had been achieved with the data. Saturation was achieved when the research team identified that no new themes/sub-themes were emerging from our analysis of the transcripts.

**Table 1:** **Demographic and professional characteristics of study participants**

	***N***	**Range**	***M(SD)***
Total*	34		
**Gender**			
Male	2		
Female	32		
**Age of Participants**	31	24-61	42.42 (10.10)
**Professional Affiliations**			
Social Work (Masters)	11		
Psychology (Masters)	10		
Psychiatry	5		
Other**	8		
**Years working in eating disorders**	31	1-26	8.40 (6.86)
**Years working with adults (over 18 years old)**	31	0-26	10.53 (7.60)
**Years working with adolescents**	31	0-27	10.52 (7.60)
**Years working with families**	31	0-27	10.34 (7.99)
**Level of FBT Training**	6	[0] no formal training or purposeful exposure	
	13	[1] some exposure to FBT, no formal training	
	9	[2] undergoing FBT certification	
	6	[3] certified FBT therapist	
**Endorsed training in other form of family therapy**	22	No	
	9	Yes	

*To maintain the confidentiality of all clinicians, the demographic data for focus group and individual interview participants are purposefully combined.

**To maintain the confidentiality of all clinicians, all professions with five or fewer representatives have been combined into the category of ‘other’.

**Table 2:** **Example questions from the semi-structured interview guide**

**Question**	**Objective**
Describe how you employ FBT in your program and specifically with adolescents and their families.	To discover how clinicians use FBT within their programs.
How do you introduce and use FBT with families across the span of adolescent development?	Gain understanding of how clinicians explain and introduce the interventions and principles of of FBT.
How is [insert intervention of FBT] used across adolescent development with families?	Discern how clinicians and treatment teams are using FBT similarly and differently with adolescents and TAY.
Probes: How and why did you adapt that intervention? How do adolescents/TAY and families respond to this adaptation?	To explore further the responses given by clinicians/ treatment teams/adolescents/TAY and families to adaptations to FBT in each phase.

### Sample

From across Ontario, clinicians who provide treatment for adolescents and TAY with AN and BN participated in focus groups (FGs, n = 6). Seven individuals participated in individual interviews. Thirty-two participants identified as women and two as men. Of the 31 clinicians who reported their age on the self-report form, the sample ranged in age from 24 to 63 with an average age of 42.42 (SD = 10.10) (Table 1). The majority of participants had a Master’s of Social Work (n = 12), with Doctorates in Psychology (n = 10) and Psychiatry (n = 5) comprising the next two largest groups. To maintain the confidentiality of clinicians, professions with five or fewer representatives have been combined into the category of ‘other’.

The sample self-identified as having extensive clinical experience: an average of 10.34 (SD = 7.99) years working with families, 10.52 (SD = 7.60) years working with patients under the age of 18, and 8.40 (SD = 6.86) years working with eating disorders. Three participants identified as being certified in FBT, 9 were currently undergoing the FBT certification process, 13 had exposure to FBT through seminars, reading materials, or team members who were trained in FBT, and 6 had no formal training or purposeful exposure to FBT. Individuals with no formal training or purposeful exposure to FBT were included in the analysis only if they were members of clinical treatment teams who identified as using FBT in their program. These participants attended focus group discussions.

### Data analysis

FGs and individual interviews were digitally audio-recorded and then transcribed verbatim by a professional transcriptionist. Any identifying information such as names and places were removed prior to data analysis. Directed content analysis [26,27] was utilized to describe and quantify how FBT is used with TAY. A series of steps were employed to conduct this type of analysis [28]. First, to prepare for the analysis, categories were pre-defined using the research question and key interventions of FBT (e.g.: *creating a sense of urgency*, *the family meal*, *session length,* and *developmental appropriateness*). A determination of categories was conducted by identifying the principles, phases and interventions outlined in the FBT manual for both AN [2] and BN [17]. Second, two members of the research team (VF and BA) conducted independent coding of the transcripts using the above pre-defined categories based on the model. Each transcript was read in full and then re-read line by line to extract pre-defined categories using codes (variations in the use of FBT with young adolescents and TAY) and a coding list. The qualitative data was re-read multiple times and codes relating to each category were defined and grouped together.

The codes were then discussed with the principal investigator (GD) to ensure that selected text accurately represented the category (phase or intervention as described in FBT) that it was grouped within. To verify the reliability of the codes generated for each category, the PI randomly selected 20% of transcripts for independent coding. Categories were created by collapsing codes with a high frequency of occurrence in the individual interviews and focus groups. Any dissonance in coding or how codes were collapsed was resolved through discussion until consensus was reached. The research team then defined each category using the description of the phases and interventions used in the FBT model. To maximize the trustworthiness of our data, the research team presented their preliminary research findings during a provincial meeting held by the Ontario Community Outreach Program for Eating Disorders in December 2013 where the majority of research participants were in attendance. This provided clinician participants and others working with TAY to indicate if the findings resonated with them.

## Results

The results are organized by the categories used in directed content analysis; the categories were defined by the research question, the interventions or phases of FBT (e.g.: *externalization, phase 1, developmental appropriateness*, etc.). Details of each intervention and phase of FBT have previously been described elsewhere [1,17]. Overall, clinicians in both the FGs and the interviews endorsed using FBT differently with TAY across all phases of FBT. For a summary of all findings, please see Table 3. Session 2 (the family meal) was not included in this analysis because minimal adaptations were discussed in the interviews and focus groups. We identify quotes from transcribed interviews below as being from Individual Interviews (I) or Focus Groups (FG) and by an anonymous participant code assigned to the study site or study participant.

**Table 3:** **Adaptations to Family-Based Treatment (FBT): Results from Individual Interviews (I) and Focus Groups (FG)**

**Intervention of FBT**	**I or FG**	**N**	**Findings of Adaptation or No Adaptation to Interventions of FBT with TAY versus Younger Adolescents**
Individual Time with the TAY	I	1	No difference with age, adaptations are based on developmental stage
		1	No individual time with TAY
		1	No time specified, but individual time with a TAY is longer
		6	Change in time spent during session with TAY – ranging from 15 minutes to the entire session spent individually with a TAY (those advocating entire session suggested reserving this for Phase 3)
		4	Content of the individual meeting with a TAY is about motivation and alliance
	FG	3	The individual time is longer with a TAY across all phases
		1	The individual time is longer with a TAY in phase two only
		1	The individual time is longer with a TAY in phase three only
		1	If individual time is extended, there is the risk of excluding the family
		3	Content of the individual meeting is about impacts of the ED on TAY; about family dynamics; and, support a TAY’s autonomy
Externalization	I	3	Externalization looks the same with a TAY
		3	TAY are more capable of abstract externalization
	FG	3	Externalization looks the same with a TAY
		4	TAY are more resistant to the idea of externalization
		1	TAY are more accepting of the idea of externalization
		3	Visual externalization is used for younger teens while TAY are insight oriented
		2	Externalization is directed even more strongly at parents of TAY
Creating an Urgent Message to Support Parents to Help their Child make Behavioural Changes	I	2	The message is the same regardless of age
		5	The message is created for both the TAY and the parents
		4	The content of the message is focused on social and future goals for TAY
		4	The transition to adult-care systems is used to increase anxiety of TAY
		4	The language of the urgent message is more explicit for TAY
	FG	6	The message is the same regardless of age
		5	The message is created for both the TAY and the parents
		5	The content of the message is focused on social and future goals for TAY
		3	The transition to adult-care systems is used to increase anxiety of TAY
Phase 2	I	5	TAY need more opportunities to practice eating independently
		3	TAY need more opportunities to practice eating at school and work
	FG	1	Phase 2 is not based on age, it simply varies from individual to individual
		2	There is more discussion of a TAY’s independence in phase 2
		2	It is more difficult to keep parents of a TAY engaged in phase 2
		1	Phase 2 is more collaborative with a TAY around meals
		1	Phase 2 is framed as training ground for post-secondary and work
		1	Parents are more likely to give back control too quickly for a TAY
Phase 3	I	6	Relapse prevention is incorporated into phase 3 for a TAY
		3	There is more talk of future oriented goals with a TAY
		2	Phase three contains individual therapy for a TAY
		3	Different issues such as body image, life transitions, future goals, and emotion regulation are discussed with a TAY
	FG	5	Relapse prevention is incorporated into phase 3 for a TAY
		3	Phase three contains individual therapy for a TAY
		3	Different issues such as body image, life transitions, future goals, and identity developmental are focused on for TAY
		2	Phase three is shorter for a TAY

Number of Interviews that Endorsed Adaptation or Non-Adaptation.

### Category 1: Developmental appropriateness for FBT

The interview began with a general question about whether participants utilized FBT in the same way with patients across the developmental phase of adolescence. The majority of the participants in the individual interviews (four of the seven) and the focus groups (five out of six) described the model as being developmentally inappropriate for TAY. Further, some participants in the individual interviews noted that parents of TAY also found the model to be developmentally inappropriate. One participant shared, “*families of young adults tend to move into phase two too quickly because [phase one] does feel so developmentally inappropriate. I think that sometimes parents want to give young adults that control back so badly but they’re just not ready*” *(I, 16).* It is important to note that participants with more extensive FBT training agreed with the participants in the focus groups regarding the developmental appropriateness FBT with TAY.

Participants across both the individual interviews and the focus groups provided similar reasons for why FBT may be developmentally inappropriate: with respect to the TAY, participants noted that the increased level of autonomy in this age group made it difficult to engage them in a model that encourages parental monitoring. For parents, participants noted their struggle to support re-nourishment given their child’s gained independence in areas outside of eating. This balance between re-nourishment and a child’s autonomy is exemplified by the following quote: “*Parents are often a little more reluctant to step in and take full control because they’ve had three, four, sometimes even five years of giving the kid more autonomy in just about every other area of their* life” *(FG 29)*. Although the majority of the participants in the interviews and focus groups endorsed that the model was developmentally inappropriate for TAY, the remaining participants noted the appropriateness for TAY especially when the affected individual still lives at home, attends high school and appears developmentally regressed due to the illness. Under these circumstances, some clinicians argued that there are few clinical differences between a younger adolescent and a TAY with an ED.

### Category 2: General differences in the application of FBT with a TAY

Participants were asked to discuss differences and similarities in the application of FBT with young adolescents and TAY in their clinical practice. According to all of the participants in the interviews, a more collaborative therapeutic approach is used with TAY than with young adolescents throughout the process of FBT. One participant shared that “*there’s more interaction with young adults because at their age they tend to have a better perspective on things. I think that they are able to, even if they can’t make choices about their food, contribute to the session in more meaningful ways than adolescents can. So even though their parents might be choosing their food, the young adults may give more input into – okay, well the parents are making the decision but I would like this, or can we do this or can we try this?” (I, 16)*. Overall, these clinicians described a collaborative approach in three distinct ways: first, clinicians recognized that while parents should intervene to acquire control over eating and physical activity when their child is ill with an ED irrespective of age, TAY must be actively engaged in negotiating with their parents *how* the behavioural changes occur on a day-to-day basis; second, it was vital to garner the explicit consent of the TAY to allow parents to support them with ameliorating eating disordered behaviours; and third, collaboration with TAY is only possible when she/he has an increased appreciation of the consequences of the illness.

Similar to the individual interviews, the majority of focus group participants (four of six) endorsed being more collaborative with a TAY. “*[TAY] are just more at the table. It is still the premise that this [re-nourishment] needs to happen … but they’re more at the table in terms of the discussion and negotiation, which feels appropriate in terms of adolescent development” (FG 7).* The focus group participants and the participants in the individual interviews provided the same reasons for modifying the application of FBT with a TAY: while parental support around meals and eating disordered behaviours were perceived as necessary, collaboration with the TAY was seen to be more developmentally appropriate and the temporary nature of parental support was explicitly stated to TAY in order to respect and recognize their discomfort in temporarily losing independence around eating. The similarities in the rationale provided in the individual interviews and focus groups demonstrate that formalized training in FBT did not deter clinicians from considering how to use this model differently with TAY.

### Category 3: Individual time with TAY prior to the meeting with the family

Participants were asked if the time spent with the patient prior to the family meeting was influenced by the age of the affected individual. With the exception of one, all clinicians in the individual interviews extended the individual time spent with the TAY. In FBT for adolescents, no more than 10 minutes are dedicated to this individual time with the patient. Although there was variability in the amount of time spent with the patient (see Table 3), participant clinicians described increasing the individual time with the patient for the following clinical reasons: TAY were seen as more capable and willing to share details of their meals/symptoms with clinicians and time spent individually was more necessary to build therapeutic alliance with TAY when compared to younger adolescents.

Among the focus groups, there was no consensus regarding whether time spent individually prior to the family meeting was applied similarly or differently with TAY. Only half of the focus groups (three out of six) endorsed a longer individual session with TAY prior to bringing the parents into the session. Clinician participants in the focus groups noted that discussions with the TAY during these individual sessions focused on how the eating disorder interferes with their life while delving into challenges experienced by the TAY with reference to how family members were supporting them (or not) with behavioural changes. Clinicians also noted that TAY are more likely than younger teenagers to speak about challenging family dynamics and stressors that impede their parents to support them with behavioural changes. Clinicians similarly discussed utilizing individual time with clinicians to reinforce the autonomy of the TAY. Of the focus groups who did not agree to adapting the amount of time spent individually with patients based on age the main concern was that this would result in reduced time with the entire family and the potential of disempowering the parents. It is interesting to note that there was more consensus about meeting for a longer period of time with a TAY with an eating disorder among the clinicians with greater training in manualized FBT, whereas there were varying views about adapting this aspect of the model based on age in the focus groups. It is possible that clinicians with greater training in FBT are less concerned about the length of the individual time with the patient because they perceive themselves as engaging in discussions with the TAY that are congruent with the model. In contrast to the individual interviews, the participants in the FGs engaged in discussions about a range of issues that slightly deviated from what is prescribed in manualized FBT (focus on family dynamics for instance).

### Category 4: Session 1

We systemically asked participants in the focus group and the individual interviews to discuss if the age of the individual with the eating disorder influences how they apply each intervention in session 1 and in each phase of FBT. Significant adaptations were described for TAY and their parents only for the following interventions in session 1: externalization, and the content of the urgent message for parental involvement in treatment. We further highlight the adaptations to the interventions for each phase below.

### Category 4a: Externalization

Externalization refers to separation of the individual from the eating disorder throughout treatment in order to reduce parental blame and promote parental empowerment. Six of the individual interviews discussed externalization based on age. It is important to note that none of the participants in the interviews endorsed adaptations to externalization, but instead endorsed differences in the application of this intervention (Table 3). For those who did note differences to externalization (3 of 6), clinicians credited the TAY’s ability to more readily understand abstract concepts and illustrations of how to separate the illness from them. “*Abstractly, older kids might be able to identify with the idea of externalization more…they might be able to relate to what I’m talking about and expand more…whereas the younger ones…it’s more the parents who are kind of grabbing onto those concepts” (I, 65)*.

Similar to the individual interviews, three focus groups reported applying the same principles of externalization, regardless of the age, and three reported differences in the application. Of the focus groups who endorsed differences in the application of externalization, all noted the use of more insight-oriented examples as opposed to visual depictions of the eating disorder and/or metaphors for TAY. This difference in the application of externalization was endorsed due to a TAY’s increased ability to articulate how the illness affects their perceptions, feelings and interactions with others. On the other hand, four out of six focus groups noted that TAY patients were more resistant to the idea of externalization, often because the eating disorder was long-standing and thus strongly linked to their sense of identity. These differences in externalization are exemplified by the following quote: *“I find [TAY] don’t like it, they get mad…your job as a teenager at that point is to figure out identity, self-concept, and someone’s kind of telling you that it’s an eating disorder that’s part of you. I think that’s an insult to some kids. You’re saying you’re not in control, there’s something that’s taking over your thinking, and I think that can be really scary” (FG 24)*.

### Category 4b: Urgent message for parents to support their child to make behavioural changes

In session 1, the most critical intervention is the “urgent message”, the delivery of information to the parents about the seriousness of the eating disorder and the urgent need that they act immediately to assist their child to gain weight and eat normally. Clinicians across the interviews unanimously endorsed adaptations to the *urgent message* [1,17] with a TAY versus a younger adolescent. First, participants in the individual interviews noted that the message of urgent action was no longer directed solely towards activating the parents to engage in promoting symptom change in their child, but was developed for both the parents and the TAY. Clinicians credited the TAY’s greater appreciation for the consequences of the illness as the rationale for delivering the message to them and not just to their parents. The second adaptation endorsed by the participants in the individual interviews was to the content of the urgent message. With a TAY, topics such as peer relationships, fertility, university, and obtaining a drivers licence were used to increase motivation to propel them into agreeing to involve their parents to assist them with making behavioural changes: “*We might talk more about future as opposed to present with older kids, like when they go off to school or the impact of this on people’s relationships” (FG 24).* Clinicians also included content about the dearth of effective treatments in the adult system of care of eating disorders for TAY under the age of 18. In Ontario, paediatric care can often not be provided beyond this age. Clinicians felt that the time-sensitive nature of treatment for a TAY under the age of 18 was particularly useful in creating a sense of urgency for the family to leverage their control over eating and meal support. Finally, the language of the urgent message was described as more “blunt” and “scary”, such as describing in great detail the severe medical consequences associated with this illness when speaking with a TAY versus a younger adolescent. In fact, clinician participants agreed that this type of strong and urgent message should only be delivered to parents of young adolescents because the goal is to empower the parents to engage in behavioural change. In contrast, with TAY the blunt and direct message about the necessity of treatment is directed simultaneously to the parents and the TAY to facilitate them working collaboratively to make changes to eating and weight.

Initially, all of the participants in the focus groups reported that the urgent message delivered to the family was the same regardless of age. However, as we further probed about this intervention, we found that all of the participants in the focus groups endorsed adaptations when delivering the urgent message to a family of a TAY. Participants agreed that the urgent message to engage in change should be delivered to the parents *and* the TAY rather than just the parents of young adolescents.

Regardless of level of training in FBT, clinicians in the individual interviews and focus groups described adhering to the principles of FBT (parental empowerment in re-nourishment, and externalization of the illness), but reported varying the message and using language deemed more developmentally appropriate for TAY. Although faithful to the model, clinicians also describe targeting their interventions directly to the TAY and their parents whereas messages about the importance of parental control over eating are delivered more exclusively to the parents of younger adolescents.

### Category 5: Phase one

The main objective of phase one is for parents to support their child to gain weight, eat normally and cease using purging methods. Adaptations to the interventions in phase one were endorsed by the participants in the individual interviews (five out of seven) and focus groups (five out of six). The majority of participants reported that they were more likely to collaboratively engage the TAY in phase one treatment in contrast to young adolescents. One participant captured the sentiment described by the clinicians in this study: *“I think [TAY] are able to contribute to the session in more meaningful ways. The parents are making the decisions but they can say ‘I would like this, or can we try that.’ I think in that way, [TAY] can give more suggestions that are coming from them and not the eating disorder whereas input from adolescents is being driven by the eating disorder” (I, 16).*

The remaining adaptation to phase 1 was only described in the focus groups (four of five) and not the individual interviews; clinicians noted a need to incorporate more psychoeducation material about the illness and explicit discussion of the rationale for having parents gain control of the re-nourishment process for TAY rather than for young adolescents.

### Category 6: Phase two

In phase two, six out of seven participants in the individual interviews and only three of the five FGs endorsed adaptations in the interventions in this phase with TAY versus a younger adolescent. The aim of phase two is to return control of eating back to the adolescent. We only report below on the interventions for which participants achieved consensus on how they should be adapted for TAY. Five of the six participants in the individual interviews endorsed that TAY should be provided with more opportunities to practice regaining independence over their meals since they are likely to have autonomy in many other areas of their lives. *“It’s normal for a young kid to have most of their meals with parents whereas an older teen wouldn’t. …In stage two there is more to give back. They are talking about having some meals on their own, they’re doing more socializing too, so that piece has to be worked on. To get them more involved with friends whereas little kids don’t do as much of that” (I, 62).* Further, the participants in the individual interviews described encouraging TAY to practice eating outside of their home, at work and in school. Finally, participants all agreed that parents should relinquish the control of eating much more rapidly to TAY than adolescents in order for them to practice preparing meals and eating with greater independence.

Within the focus groups, no adaptations were consistently endorsed for phase 2. The wide variety of adaptations can be viewed in Table 3.

### Category 7: Phase three

The overarching objective of phase three is to explore issues pertaining to adolescence and to identify how normative adolescent changes may affect recovery. Clinicians in the individual interviews and the focus groups endorsed one consistent adaptation to FBT with a TAY in phase three. Six of the clinicians in the individual interviews and five of the focus groups described incorporating relapse prevention into phase three work with a TAY, but rarely doing this with young adolescents. *“I find with younger ones that there isn’t a whole lot to talk about in phase three. With older ones…we focus on ‘what is your new way to be without the ED?’ ‘How are you going to extricate this thing from your identity?’ ‘How do we know this isn’t going to come back’ and what guarantee can we put on that” (I, 44).* Overall, clinicians described the importance of TAY planning in advance for multiple life transitions such as university/college, independent living and career choices with special attention paid to how to prevent slips and relapses and when to seek parental and professional support.

In summary, clinicians in both the individual interviews and focus groups described adaptations to the delivery of FBT with a TAY and their family when compared to the delivery of FBT with a younger adolescent. When adaptations were endorsed, clinicians frequently asserted that they were made due to the increasing autonomy and independence of TAY when compared to younger adolescents. Finally, exposure to training and certification in FBT did not lead to fewer adaptations to this model based on age. In fact, there was often consensus between the clinicians in the individual interviews (who had more FBT training) and focus groups about modifying how FBT is delivered with TAY.

## Discussion

The purpose of this study was to systematically identify if and how the principles and interventions of FBT differ when working with young adolescents versus with TAY. Following a structured interview guide, we inquired about possible adaptations to FBT interventions in session 1, phase 1, phase 2 and phase 3 in the provision of care for TAY compared to adolescents. Overall, the findings of this qualitative analysis were congruent with the recommendations of the Family-Based Treatment manual for AN [2] and BN [18], which state that the clinical interventions should be tempered based on the presentation of the patient, including factors such as age and developmental stage. The findings of this study can be categorized into two themes. First, clinicians in the interviews and FGs consistently endorsed adaptations to FBT when working with TAY compared to young adolescents. The age of individuals with eating disorders, their independence in many areas of their lives including meals, and their pending transfer of care from paediatric to adult eating disorder programs are factors that influence the decision to adapt the principles and interventions of FBT. Second, the number of adaptations to FBT progressively increases over the course of treatment when working with TAY in contrast to younger adolescents with eating disorders.

The findings of this study demonstrated that clinicians consistently adapted specific interventions across the different phases of FBT when working with TAY. Participants increased the amount of time spent with TAY compared to younger adolescents prior to beginning their session with the whole family. Even when clinicians reported closely following manualized FBT by only meeting briefly with the patient to weigh him/her, some participants agreed that the content of these individual meetings differed because of the age and development of the patient. For example, clinicians described using individual time with TAY to enhance their engagement in the treatment process. This is consistent with previous literature which has described the importance of engaging older adolescents and TAY in FBT [33-35].

Along the same lines as engaging TAY throughout the family-based treatment, most of the clinicians described working to establish a collaborative relationship with the patient, particularly in the first phase of treatment. To promote collaboration, clinicians described that they simultaneously worked to empower parents and enlist the involvement of the patient in temporarily acquiescing to the efforts of their parents to re-nourish them back to health. Although clinicians reported a preference to follow manualized FBT, they also acknowledged collaborating with TAY during FBT more frequently than younger adolescents because developmentally, older adolescents demand and insist on greater independence and autonomous decision-making in many areas of their lives, including eating and meals. However, the participants in this study also acknowledged the necessity of parental involvement in facilitating behaviour changes and reducing eating disorder symptoms since the patient, regardless of age, cannot fully take responsibility for recovery due to starvation and emaciation particularly in the early stages of treatment. The importance of parental involvement in eating disorders in TAY across this developmental phase has been demonstrated in other research that shows parental monitoring reduces instances of high-risk behaviours in this age group (16–21), including substance use, sexual activity and violence [35].

The second finding that emerged from this qualitative analysis is that adaptations to manualized FBT increase over time more significantly when working with TAY in contrast to younger adolescents. Adaptations represent a departure from the foundational, manualized approach, and may contribute to a decline in fidelity to FBT that has been demonstrated in previous studies [15]. However, clinician perspectives and reasons for decreasing their adherence to FBT in the later phases of treatment have not previously been investigated. With respect to phase 2 (transferring parental control of the eating to the adolescent), clinicians uniformly agreed that TAY must have ample opportunities to practice eating meals on their own and in various social contexts (i.e., work and school) and with individuals external to the family. The majority of clinicians reported making the greatest adaptations from the foundational model of FBT in phase 3. All of the participants in the interviews and the majority of the participants in the FGs indicated that they met *individually* with the TAY in phase 3 to address body image concerns, self-esteem issues and relapse prevention. The incorporation of cognitive therapy and relapse prevention in the later phase of FBT was consistently used to assist patients to maintain behavioural changes that chiefly occurred because of parental involvement and monitoring.

### Implications for clinical practice and future research

This qualitative study demonstrates that clinicians appear to maintain the overall philosophy of the FBT model when working with TAY with eating disorders and their families, but do make some adaptations. Firstly, clinicians working with TAY emphasized engaging the patient from the very outset of FBT, although they acknowledged the importance of parental support for making behavioural changes such as eating rehabilitation and weight restoration. Most clinicians advocated taking this a step further and adopting a collaborative approach, which they perceived as being more developmentally appropriate. For example, a collaborative approach would provide greater opportunities for TAY to practice eating in various contexts and with increased independence from their parents. A second type of adaptation reported in this study was that clinicians see older youth individually and incorporate more relapse prevention and CBT principles in the final phase of the model. A recent open trial has investigated CBT as an alternative to FBT [33], but no controlled trials have been published. To date, there are no longitudinal studies evaluating the effectiveness of CBT following the completion of FBT although it has been described and recommended [36,37].

Future research efforts should begin with the development of treatment manuals that integrate conventional FBT with the changes identified in this qualitative study, following which the manualized treatment can be systematically evaluated. To date, there is only a single published case series [24] of FBT adapted for TAY, and more research is clearly needed including a randomized controlled trial to evaluate FBT adapted for TAY. Our group is currently conducting a feasibility study of FBT adapted for this age group.

### Strengths and limitations

This study, the first to our knowledge to qualitatively examine the perspective of clinicians on how FBT is used similarly and differently when working with TAY compared to young adolescents, has a number of strengths. First, an interview guide was developed using the principles and interventions described in FBT manuals for AN and BN. The same interview guide was used consistently in all of the interviews and FGs to maintain standardization with the questions employed. Second, a heterogeneous group of clinicians with varying clinical backgrounds and disciplines participated in this study. The views of interdisciplinary teams were solicited as well as the experiences of individual clinicians with training in FBT; separate interviews were conducted with individuals who had extensive clinical experience with FBT and/or were certified therapists/supervisors. Finally, another strength of this study is that clinicians were interviewed from across the Province of Ontario to ensure that the perspective of clinicians working in both rural and urban areas and from programs with varying levels of resources were captured.

This study has a number of limitations that are important to highlight. It is possible that participants in a focus group had greater difficulties speaking openly about not always adhering to an empirically supported model in a treatment program that promotes itself as utilizing evidence based practice models. However, a careful comparison of the individual interviews and focus groups did not reveal significant differences about adaptations to the model. This study relied on the perceptions of clinicians regarding their modifications to FBT for adolescents when working with TAY and therefore is subject to recall bias. Another potential limitation to this study is that the clinicians who participated did so voluntarily and therefore may have different characteristics than the clinicians who did not participate. Results may also be difficult to generalize to other settings given that the participants had access to ongoing training in the treatment of eating disorders and worked within a publicly funded health care system, setting them apart from clinicians working in privately funded settings and in other provinces or countries.

## Conclusions

FBT is recommended for young people across the developmental stage of adolescence and TAY. Through the use of focus groups and individual interviews, clinicians reported adapting many of the principles and interventions of FBT when working with TAY. These adaptations were reported regardless of the degree to which participants had received formalized training in FBT. Clinician participants described making adaptations to the model in recognition of the propensity for older adolescents to have greater autonomy and independence and resist complete parental control over eating without securing some agreement on the part of the TAY. Similarly, clinicians remained adherent to the core principles of this model by empowering parents to continue to support their child regardless of age with the goals of achieving recovery from an eating disorder. Greater opportunities to practice eating with parental support were discussed for phase 2 and an introduction of relapse prevention strategies in the final phase of this model were noted. Improved understanding of how this treatment can be adapted for TAY will ensure that this empirically supported treatment for AN is delivered effectively and improves the lives of young people during this crucial developmental phase.

## Abbreviations

AN: Anorexia Nervosa
BN: Bulimia Nervosa
CBT: Cognitive Behavioural Therapy
FBT: Family-Based Treatment
FBT-TAY: Family-Based Treatment for Transition Age Youth
FG: Focus Group
I: Individual Interview
OCOPED: Ontario Community Outreach Program for Eating Disorders
TAY: Transition Age Youth
